# Quasi-Orbital Angular Momentum (Q-OAM) Generated by Quasi-Circular Array Antenna (QCA)

**DOI:** 10.1038/s41598-018-26733-6

**Published:** 2018-05-30

**Authors:** Reham M. Fouda, Thomas C. Baum, Kamran Ghorbani

**Affiliations:** RMIT University, School of Engineering, Melbourne, 3000 Australia

## Abstract

Orbital Angular Momentum (OAM), as a property of Electromagnetic (EM) fields has recently been proposed for Radio and Microwave communications. This paper investigates a new class of OAM radiation patterns for Radio and Microwave applications, namely, Quasi-OAM radiation patterns, induced by a proposed Quasi-Circular Array Antenna (QCA). Simulations and Experiments show that Quasi-OAM waves can be induced and preserved in the far-field using the proposed QCA apertures and configurations, demonstrating non-integer dominant OAM modes corresponding to *l* = +1 and *l* = −1 with a directional quasi intensity and rotational 2π phase profiles. The proposed method in this work significantly reduces aperture size and cost by using Quasi-Circular Arrays of *N*_*Q*_ = 5 and *N*_*Q*_ = 6 elements in lieu of conventional OAM circular aperture arrays with N = 8 elements.

## Introduction

In response to ever increasing high demands for larger channel capacities, EM spectrum efficiency, and communication security, several technologies have been introduced, such as Multiple-Input Multiple-Output (MIMO)^1^, Spread-Spectrum^2^, Multi-level modulation^3^ along with others. Another recently proposed method to address higher channel capacity, more robust anti-jam capabilities, spectral efficiency for next generation wireless systems and possibly next generation cellular 5G networks^4^ stems from optical studies of light behaviour. Recognising fundamental EM properties of light such as Angular Momentum (AM) and its Orbital Angular Momentum (OAM) field component as additional characteristics of electromagnetic fields which would later make it possible to exploit in Radio and Microwave communications, addressing some of the wireless community demands^5–7^.

Only recently has OAM been suggested for radio communications^8^, attracting ample interest in the use of OAM in lower frequencies than the optical regimes, and suggested as a solution providing spectral efficiency, higher channel capacity^9,10^ and anti-jam capabilities^11^. This attention however, has not been without speculation and criticism, where some in the research community have claimed that OAM is an implementation of MIMO communications^12^. While pioneers of OAM in Radio argue that through experimentation and confirming theoretical predictions by Abraham over a century ago^13^, OAM physical properties are inherently different from engineered techniques such as MIMO. Thus, the two are conceptually incompatible and cannot be directly compared^14^. Within the Microwave region of the electromagnetic spectrum, research in OAM has mainly focused on methods to generate OAM radiation patterns. OAM applications such as Radar to achieve super-resolution for target detection^15^ and Secure Multiplexed Communications have been proposed^6,9,16–18^. Circular antenna arrays (CAA)^19^ have also been proposed as one of the methods used to generate OAM radiation patterns. Such arrays can be realised by incorporating *N* equidistant elements fed with the same signal and amplitude, whilst circumferentially varying their phase. Other methods of OAM radiation pattern generation include staircase Spiral Phase Plates (SPP)^20^, helicoidal parabolic antenna^17^, phased patch arrays^21^, Reconfigurable Uniform Circular Arrays (UCA)^5^ and Circular Time-Switched Arrays (TSA)^22^. These examples further highlight that OAM radiation patterns do not always require array topologies to be generated, such is the case with MIMO systems.

Although the first experiment to generate an OAM radiation pattern at microwave frequencies was performed in a lab setting in 1994^23^, the experiment which inspired many in the Radio and Microwave research domains to look into different methods of generating OAM radiation patterns was the first outdoor demonstration of an OAM-based transmit and receive radio communication system performed in Venice, 2012^17^. In this experiment, two independent simultaneous OAM-based channels were transmitted on the same frequency over a distance of 442 m. This experiment indicated that OAM beams remain orthogonal in the far-field region and can enable the transmit and receive of signals on the same frequency.

One of the main challenges in OAM-based communication systems is the inability to transmit over very long distances due to beam divergence^16^. This is a more significant challenge at lower frequencies, thus, making it difficult to measure the beam’s topological charge with practical sized systems, rendering the unique OAM mode identification a challenge in long distance communication systems. Optical Free-Space OAM-based transmission systems however, were able to reach greater distances (up to 100 km^24^) as divergence is not as prominent than those seen at microwave frequencies. It was also noted that the decay rate of OAM fields intensity is dependent on their mode^25^ due to annular spatial distributions. An issue can also arise if the beam’s null vortex region, which appears at boresight, becomes too large in the deep far-field, to make an effective OAM receive array^19^. Hitherto, methods to address these issues recommend the increase of array aperture size in order to decrease the vortex size (also results in more antenna gain)^26^ and the use of partial aperture sampling techniques to compensate for a full array aperture to address beam divergence^27–29^. However, these methods inevitably increase OAM generating aperture size and cost. Therefore, to compliment OAM partial aperture receive systems^27^, where the receiver is a partial receive array, this paper proposes the utilisation of a proposed Quasi-Circular Array (QCA) transmitter for the generation of Quasi-OAM waves and a study of the generated Q-OAM waves characteristics associated with their extrinsic mode. This would therefore enable the overall reduction of OAM transmit array size and cost in OAM communication systems.

**Figure 1:**
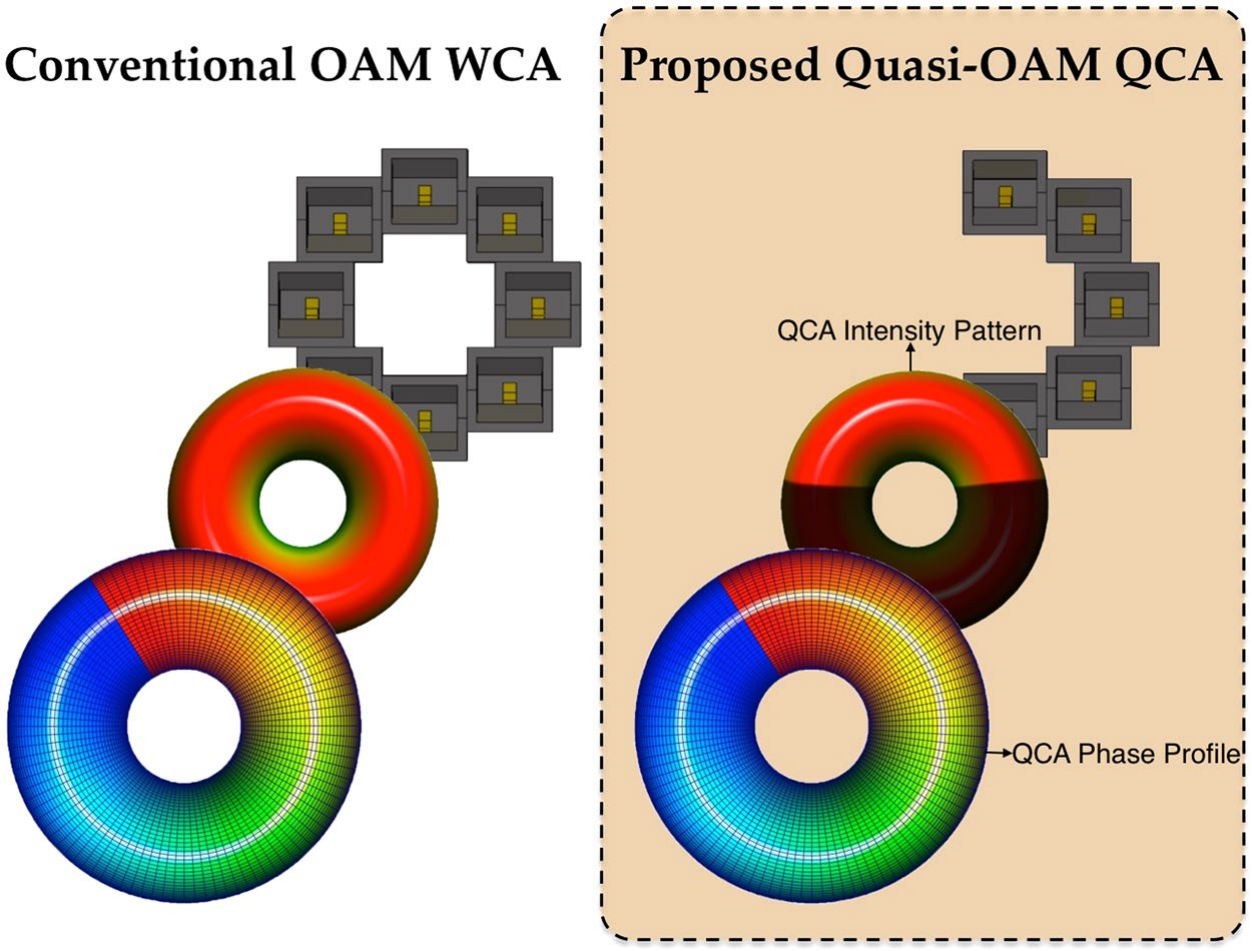
Proposed Quasi-OAM System composed of a Quasi-Circular Array Antenna generating Quasi-OAM radiation patterns vs. Conventional whole OAM system. The proposed Quasi-OAM system induces Orbital Angular Momentum in the Electromagnetic field with only a partial aperture with a partial total phase shift, resulting in a Quasi radiation pattern intensity and *l* phase profile associated with the configured OAM mode order.

In this paper, we propose a novel Quasi-OAM system using Quasi-Circular Arrays (QCA) as illustrated in Fig. 1. Whilst significantly reducing the aperture size of the arrays for practicality, the proposed QCAs of *N*_*Q*_ = 5, 6 elements are reported to produce OAM radiation patterns associated with dominant OAM mode *l* = ±1 with a higher gain of approximately ~2.0 dB to 2.6 dB as compared to conventional OAM circular array (CA) of *N* = 8 elements. Simulation and experimental results also show that the proposed Q-OAM radiation pattern results in a focused beam, as compared to the non-directional ‘doghnut’ shape of conventional OAM radiation patterns. Although the implementation of QCA apertures provides practicality with reduced apertures and cost, the authors also acknowledge that reducing the aperture’s number of elements has a direct impact on the mode power of an OAM beam, encouraging further research on cross-talk mitigation for QCA implementations and OAM mode augmentation.

## Results

### Theoretical Rationale

It is well known that EM fields carry linear momentum which is associated with translational dynamics^30^, while in 1909, Poynting predicted that circularly polarised light can also carry Angular Momentum^31^. Angular Momentum is comprised of Spin Angular Momentum (SAM) and Orbital Angular Momentum (OAM). The SAM component of AM is associated with the intrinsic polarisation of the wave, whereas the OAM component is associated with the wave’s extrinsic rotational dynamics. OAM however was not widely recognised until 1992, when Allen *et al*. recognised that light radiation patterns with a helical phase profile carries Orbital Angular Momentum^32^. Angular Momentum in electromagnetic theory is defined in Eqs (1–3)^33^:

1 $${J}_{total}=S+L$$

where

2 $$S={\varepsilon }_{0}\int Re\{{E}^{\ast }\times A\}dV$$

and

3 $$L={\varepsilon }_{0}\int Re\{{E}^{\ast }[(r\times \nabla \mathrm{)].}A\}dV$$

where *S* represents the SAM component associated with a wave’s polarisation and *L* represents the OAM component associated with a wave’s rotational phase profile. In cylindrical coordinates, the electric field at a receiving point (*ρ*, *φ*, *z*) can be described as^34^:

4 $$\begin{array}{rcl}{E}_{l}(\rho ,\phi ,z) & = & \frac{{\alpha }_{0}{j}^{l}}{\sqrt{{\rho }^{2}+{z}^{2}}}{J}_{l}(\frac{k{D}_{\rho }}{\sqrt[2]{{\rho }^{2}+{z}^{2}}}){e}^{-jk\sqrt{{\rho }^{2}+{z}^{2}}}{e}^{-jl\phi }\\  & = & {A}_{l}(\rho ,z){e}^{-jl\phi }\end{array}$$

where *α*_0_ contains the antenna constants, *k* is the wavenumber, *D* is the aperture size of the transmitting antenna, *J*_*l*_(*x*) is the Bessel function of the first kind of order *l*, *ρ* is the radial position (radius), *φ* is the transverse azimuthal angle, *z* is the transmission distance and *l* is the topological OAM mode number. As can be seen in Eq. (4), the unique characteristic of OAM waves is their azimuthal phase dependency of *exp*(*ilφ*) where each photon carries an OAM of *lħ*, which is responsible for rotating the phase profile of an OAM carrying wave.

In accord with Huygen’s principle^35^, each radiating point on the aperture of an antenna placed annularly will produce secondary wavelets which will radiate and interact with other secondary wavelets to produce constructive or destructive interference. Thus, having a varying phase to produce OAM waves will have for each point where the phase is *φ* an adjacent point with phase *φ* + *π*.

Therefore, if we place a constraint on the transverse azimuthal angle *φ* in *A*_*l*_(*ρ*, *z*)*e*^−*jlφ*^ of the electric field, such that we reduce the radiation points and adjacent wavelet interaction, the field would experience reduced destructive interference. The result is an increase in radiated gain whilst remaining orthogonal, as it still possesses an azimuthal dependence of *exp*(*ilφ*) where the overall azimuthal field distribution is *φ* = 0 → 2*π*. If we define two Q-OAM waves, ${E}_{Q{l}_{1}}$ and ${E}_{Q{l}_{2}}$ with different *l* mode numbers, such that:

5 $$\begin{array}{rcl}{E}_{Q{l}_{1}}(\rho ,\phi ,z) & = & {A}_{Q{l}_{1}}(\rho ,z){e}^{-j{l}_{1}\phi }\\ {E}_{Q{l}_{2}}(\rho ,\phi ,z) & = & {A}_{Q{l}_{1}}(\rho ,z){e}^{-j{l}_{2}\phi }\end{array}$$

therefore, we would still be able to achieve inter-modal orthogonality integrating over *φ* = 0 → 2*π* for a Quasi-OAM beam with Q-OAM modes as such:

6 $${\int }_{0}^{2\pi }{E}_{Q{l}_{1}}{E}_{Q{l}_{2}}^{\ast }d\phi =\{\begin{array}{c}0\\ {E}_{Q{l}_{1}}{E}_{Q{l}_{2}}^{\ast }\end{array}\begin{array}{c}\,{\rm{if}}\,{E}_{Q{l}_{1}}\ne {E}_{Q{l}_{2}}\\ \,{\rm{if}}\,{E}_{Q{l}_{1}}={E}_{Q{l}_{2}}\end{array}$$

and thus, Q-OAM modes are theoretically expected to be mathematically orthogonal if their inner product is zero for any two Quasi modes, ${E}_{Q{l}_{1}}$, ${E}_{Q{l}_{2}}$ using the mathematical quantisation in Eq. (6) of the well-known functional space orthogonality conditions, ensuring the ability of spatially and temporally coinciding fields to coexist, as was proven for whole OAM radiation patterns by Thidé *et al*. theoretically^36^ and experimentally^17^.

### Proposed Quasi-Circular Antenna Array Configuration for Quasi-OAM Radiation Pattern Generation

**Figure 2:**
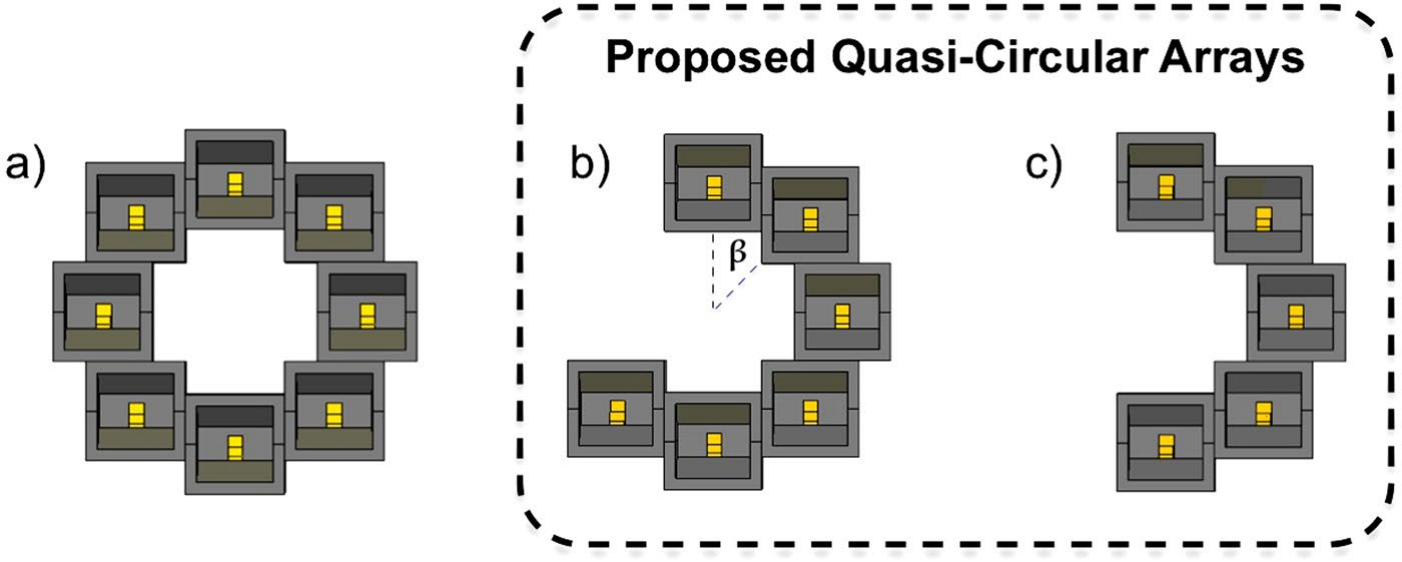
Design of (**a**) CA aperture for generating conventional whole OAM radiation patterns. Proposed QCA apertures of 6 and 5 elements in (**b**) and (**c**) respectively for Quasi-OAM generation.

Circular antenna arrays^19^ as seen in Fig. 2(a) are considered radiating elements for generating OAM as they provide OAM mode diversity. Circular Arrays can generate multiple OAM modes with *N* equidistant elements fed with the same signal and amplitude, whilst circumferentially varying the phase of each element, with an inter-element phase shift given in Eq. (7).

7 $$\delta \phi =2\pi l/N$$

where *l* is the OAM mode number, and *N* is the number of elements in the array. The theoretical number of whole OAM modes a circular array can generate^8^ is given in Eq. (8).

8 $$-\frac{N}{2} < {l}_{max} < \frac{N}{2}$$

**Figure 3:**
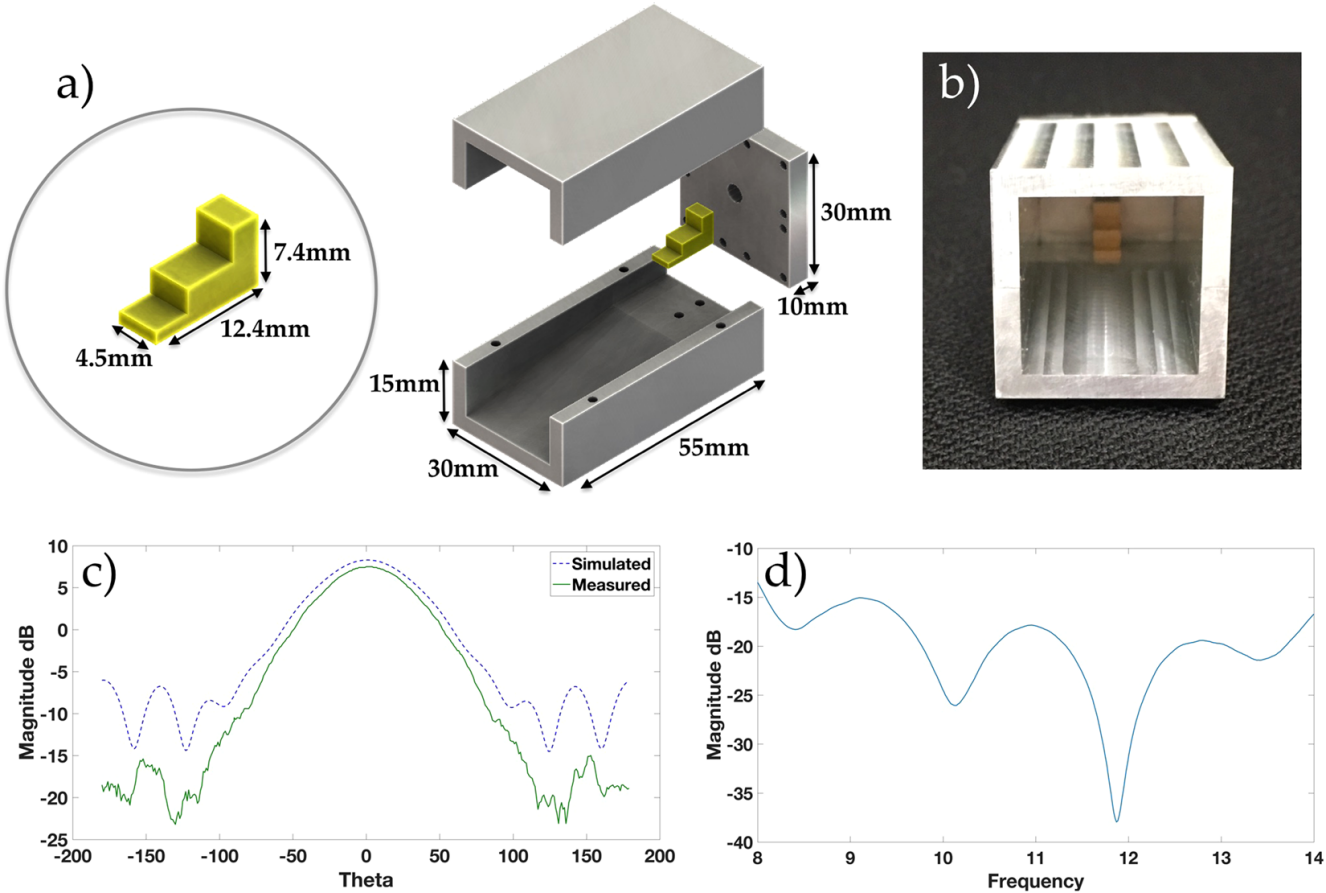
Exploded view and dimensions (**a**) of back-launched horn antenna used in the CA and QCA apertures. Front facing view (**b**) of the back-launched horn antenna. The measured and simulated radiation pattern (**c**) of the horn antenna, and measured S11 performance (**d**).

For the purpose of demonstrating the feasibility of generating Q-OAM radiation patterns, back-launched horn antenna element was simulated and fabricated for the proposed quasi-circular antenna array. The horn antenna element has a WR90 waveguide cavity with a stepped feed to enable an end-launched radiation^37^. For analysis purposes, a circular array (CA) was also built out of the same horn antenna elements to examine the generated Q-OAM radiation patterns in comparison with W-OAM radiation patterns.The back-launched horn antenna was designed to operate in the X-band region (8.0–12.0 GHz), with a measured gain of 7.5 dBi at the operating frequency 8.5 GHz. See Fig. 3(a) for the dimensions of the horn antenna, and Fig. 3(b) for its front facing view. Figure 3(c,d) display the horn antenna radiation pattern and *S*_11_ performance respectively.

As the proposed QCA apertures are restricted angular apertures, we propose the following theoretical configuration in the form of an equation. The physical inter-element angle *β* will be the main indicator of the minimum number of elements required in a QCA for Q-OAM generation, as the aperture is no longer a 2*π* aperture. This is in order to satisfy Berry’s theoretical total non-integer vortices strength^38^ to achieve mode stabilisation when the |*δϕ*| ≥ *π* (ie; half-integer). Therefore, the QCA’s number of elements *N*_*Q*_ with angle *β* between each element must satisfy Eq. (9). Aperture design of the proposed QCA apertures is shown in Fig. 2(b,c).

9 $${N}_{Q}\ge \frac{\pi }{\beta }$$

The proposed QCA is comprised of *N*_*Q*_ equidistant antenna elements on an annular arc, and fed with the same signal, uniform amplitude and a consecutive phase shift $\delta {\phi }_{Q}$ between each element given in Eq. (7). The maximum OAM mode number that can be generated by the proposed QCA configuration is the equivalent of a CA generating whole OAM radiation patterns, as derived in Eq. (10). Therefore, the smaller the angle *β* is between two consecutive elements becomes (also resulting in a higher number of elements *N*_*Q*_), the greater the number of OAM modes which can be generated.

10 $$-\frac{\pi }{\beta } < {l}_{{Q}_{max}} < \frac{\pi }{\beta }$$

The above Equations (7–10) when applied to a QCA topology, will correspond to non-integer OAM mode, with a dominating topological charge *l*. Non-Integer (or fractional) OAM modes generated by the proposed QCA apertures are superpositions of integer topological charges^20^ coexisting within the radiated beam. It is considered natural for radio and microwave frequency OAM radiation to result in a non-integer topological charge, as compared to pure OAM modes (integer *l*) generated by Laguerre-Gaussian beams^39^. Therefore, in a radio or microwave OAM or Q-OAM radiation pattern, the dominant mode becomes the beam’s topological charge. As will be seen in simulation and experimental results, the dominant mode for QCA of *N*_*Q*_ = 5 and *N*_*Q*_ = 6 is equal to *l*, satisfying Eq. (10).

The above configuration and aperture design should satisfy our theoretical analysis by preserving the azimuthal phase dependency of *exp*(*ilφ*) (which is responsible for the rotating phase fronts and orthogonality), while reducing the number of radiating elements, in order to reduce the magnitude of internal destructive interference. Consequently, this is expected to increase the radiated Q-OAM array’s overall gain compared to a conventional OAM circular array for the same *β* and *δφ*. It will also result in a reduced antenna array aperture compared with conventional CA.

### Simulation Results

Using CST Microwave Studio simulation software, we present our simulation results for two OAM modes; *l* = −1, +1 using two proposed QCA apertures. The results are compared to the whole OAM radiation pattern generated by the conventional circular array aperture. The two proposed QCA apertures illustrated in Fig. 2(b,c) are composed of *N*_*Q*_ = 6,5 elements. Each element is a back-launched horn antenna, as seen in Fig. 3. To generate a whole OAM radiation pattern, we use a whole circular aperture comprised of *N* = 8 elements, with a max diameter of *D* = 110 mm. The simulation and experiment operating frequency is set to *f* = 8.5 GHz.

Both first order OAM modes, *l* = −1 and *l* = +1 with their corresponding simulated radiation, phase, and E-Field patterns will be investigated for the CA and then the proposed QCA. For OAM *l* = +1, the total phase shift for a conventionally configured CA generating whole OAM radiation patterns is 360°, where the incremental phase shift between each element $\delta {\phi }_{Q}={45}^{\circ }$. The total phase shift for QCA apertures consisting of 6 and 5 elements is 270° and 225° respectively, with an incremental phase shift between each element $\delta {\phi }_{Q}={45}^{\circ }$, in agreement with Eq. (7). For OAM *l* = −1, the conventional CA generating whole OAM radiation patterns and proposed QCA apertures have the same total phase shift as in *l* = +1 described above, with an incremental phase shift between each element $\delta {\phi }_{Q}={45}^{\circ }$. The amplitude *A* = 1 is kept uniform across all elements and apertures, in agreement with the configuration discussed earlier in this paper. All array apertures have an angle *β* = 45° between each element. E-Field and Phase planes in the simulation results are observed from a distance of 300 mm away from each array to ensure the observation plane captures the full E-Field rotation and Phase profile away from the null region.

**Figure 4:**
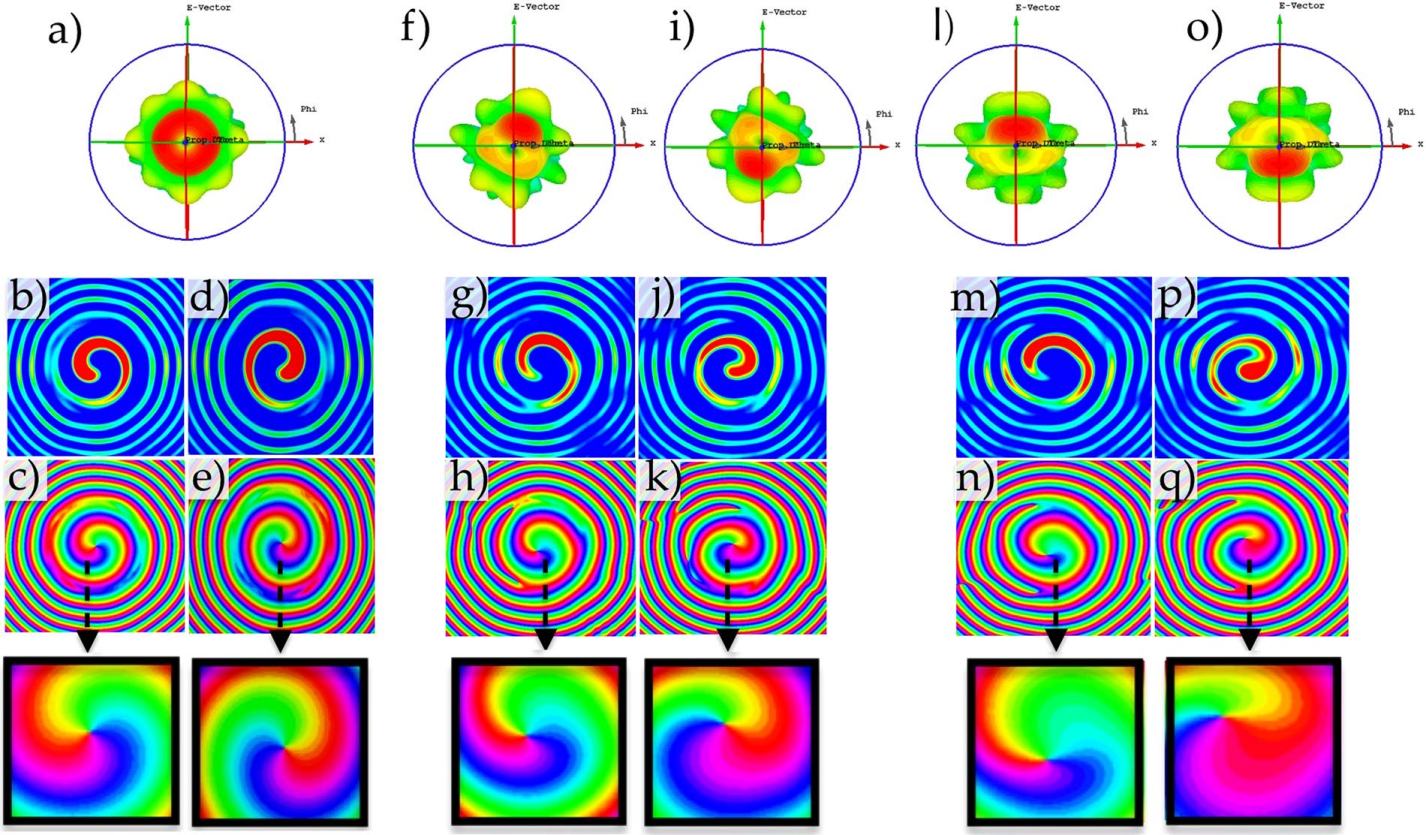
Simulated radiation pattern, E-field and phase profile of CA (*N* = 8 elements) for OAM *l* = +1 in (**a**–**c**) respectively, and OAM *l* = −1 in (**d**,**e**) respectively. Radiation pattern, E-field and phase profile of QCA (*N*_*Q*_ = 6 elements) for OAM *l* = +1 in (**f**–**h**) respectively, and OAM *l* = −1 in (**i**–**k**) respectively. Radiation pattern, E-field and phase profile of QCA (*N*_*Q*_ = 5 elements) for OAM *l* = +1 in (**l**–**n**) respectively, and OAM *l* = −1 in (**o**–**q**) respectively.

As can be seen from Fig. 4(a–e), that a conventionally configured CA produces a whole OAM radiation pattern and phase profile associated with OAM *l* = +1 and *l* = −1. The E-Field plane shows a rotating E-Field pattern in the clockwise direction for *l* = −1 and anticlockwise direction for *l* = +1. This is a characteristic of positive and negative OAM modes, and will be evaluated in our proposed Quasi OAM array to ensure corresponding mode signs. It is apparent from Fig. 4(f,l) that the QCA of 5 elements has a wider beam intensity profile than the QCA of 6 elements aperture. This is due to the fact that there are less adjacent radiating apertures. Furthermore, the more elements we place on a QCA, the more focused a beam becomes due to coherent summing. Thus the radiation pattern intensity becomes more intense in agreement with the theoretical prediction made in our theoretical rationale. Also apparent from Fig. 4(f,i,l,o) is that the vortex is present in all the radiation intensity patterns generated by the QCA apertures. This is even though the maximum intensity is only on a partial azimuthal portion of 2*π*, which is an inherit characteristic of all OAM EM waves. The phase profiles of the corresponding QCA generated radiation patterns shown in Fig. 4(h,k,n,q) display a clear rotational phase front for both proposed QCA apertures. The phase change is also proportional to the azimuthal range of the radiation pattern with respect to a full rotation of 2*π*. It also appears that by inducing a Quasi OAM wave with the QCA aperture, a full rotation in the electromagnetic field with a directional beam can be achieved. This means we can induce electromagnetic orbital angular momentum without having a full array of 2*π* radiating apertures.

To further demonstrate the rotational nature of the generated Q-OAM radiation patterns, Supplementary files S1, S2 and S3 are provided to demonstrate the rotational nature of the E-Field for Quasi OAM mode *l* = +1 in the near field, at a distance of 300 mm (~8.5*λ*) away from the CA of *N* = 8 elements, and the proposed QCA apertures of *N*_*Q*_ = 6,5 elements respectively. It is evident from the *y* component of the E-Field along with the phase profile captured in Fig. 4(h,n) and, that the Q-OAM radiation pattern generated by the QCA apertures rotate around the propagation axis in an anticlockwise direction, which is a characteristic of conventional OAM *l* = +1 mode^40^. Comparing the E-Field phase profiles in Fig. 4(g,j,m,p) with that of the full OAM radiation patterns in Fig. 4(b,d), it is evident that QCAs can induce a similar rotational phase profile to that of a CA. Hence, it is feasible to generate OAM carrying waves with a directional radiation pattern.

#### Simulated Gain Increase from Proposed QCA Apertures

**Figure 5:**
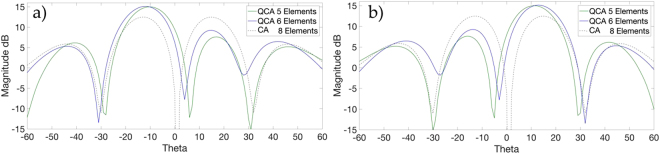
Simulated OAM *l* = −1 (**a**) and OAM *l* = +1 (**b**) radiation pattern for QCA apertures of *N*_*Q*_ = 5, 6 elements vs. CA of *N* = 8 elements showing proposed QCA gain increase.

In our theoretical rationale, we derived that theoretically, if we decrease the number of radiating points on an annular aperture, we should witness less destructive interference, and therefore an expected increase in array’s gain. Our simulation results verify this prediction for both proposed QCA apertures containing *N*_*Q*_ = 5, 6 number of elements, as compared to *N* = 8 for a conventional CA. Figure 5(a) displays the radiation patterns of all array apertures for OAM *l* = −1 and Fig. 5(b) OAM *l* = +1 for comparison. Simulation results show a 2.3 dB gain increase using QCA of *N*_*Q*_ = 5 elements, and a gain increase of 2.6 dB where QCA of *N*_*Q*_ = 6 elements is used as compared to a conventional CA with *N* = 8 elements for both OAM modes *l* = +1, −1 with a slight increase in gain for mode order *l* = +1.

This gain increase however is not to be confused with mode power, since restricting the aperture to less than 2*π* inevitably results in an increase of other modes being generated, as the resultant mode is not a pure integer mode (non-integer mode)^41^. With other modes being introduced in the generated beam, although the overall power of the beam is increased with reduced elements, the intended mode is expected to decrease in mode power. This is an important realisation which could pose as an issue in OAM multiplexed systems, and methods to augment the intended mode to decrease cross-talk is encouraged to be investigated further.

#### Quasi-OAM Azimuthal Beam Direction

The observed azimuthal beam angles of generated Q-OAM radiation patterns in simulations is dependent on the angle of the QCA center position with respect to the vertical *y* axis north. This is later verified through experimental results.

**Figure 6:**
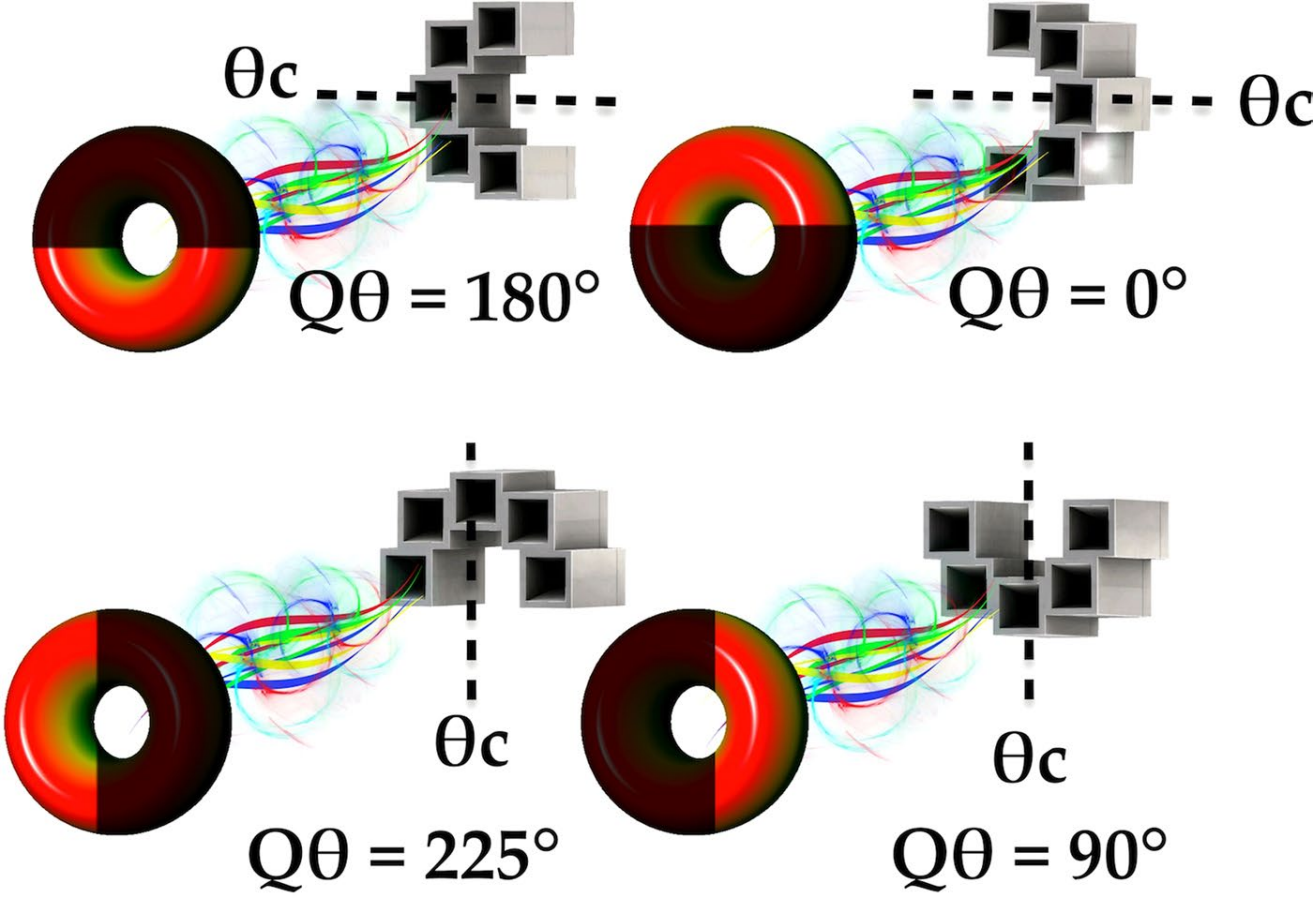
Relationship of the center position angle *θ*_*c*_ of a QCA to the azimuthal Q-OAM beam direction, where the maximum intensity of a Q-OAM beam occurs at *Q*_*θ*_, and can be derived from Eq. (11). The phase profile consequently will become a Quasi phase profile corresponding to the azimuthal angle of maximum intensity.

The center angle of the QCA *θ*_*c*_ will determine the beam’s direction, and can be derived using Eq. (11).

11 $${Q}_{\theta }=\{\begin{array}{cc}{\theta }_{c}-\frac{\pi }{2} & {\rm{if}}\,l > 0\\ {\theta }_{c}+\frac{\pi }{2} & {\rm{if}}\,l < 0\end{array}$$

where *Q*_*θ*_ is the radiated Q-OAM azimuthal angle with respect to the QCA’s *y* axis in the clockwise direction. Angle *θ*_*c*_ is the angle of the center position of the array’s arc length in degrees. See Fig. 6 for the location of the aforementioned positions and angles.

### Experimental Results

**Figure 7:**
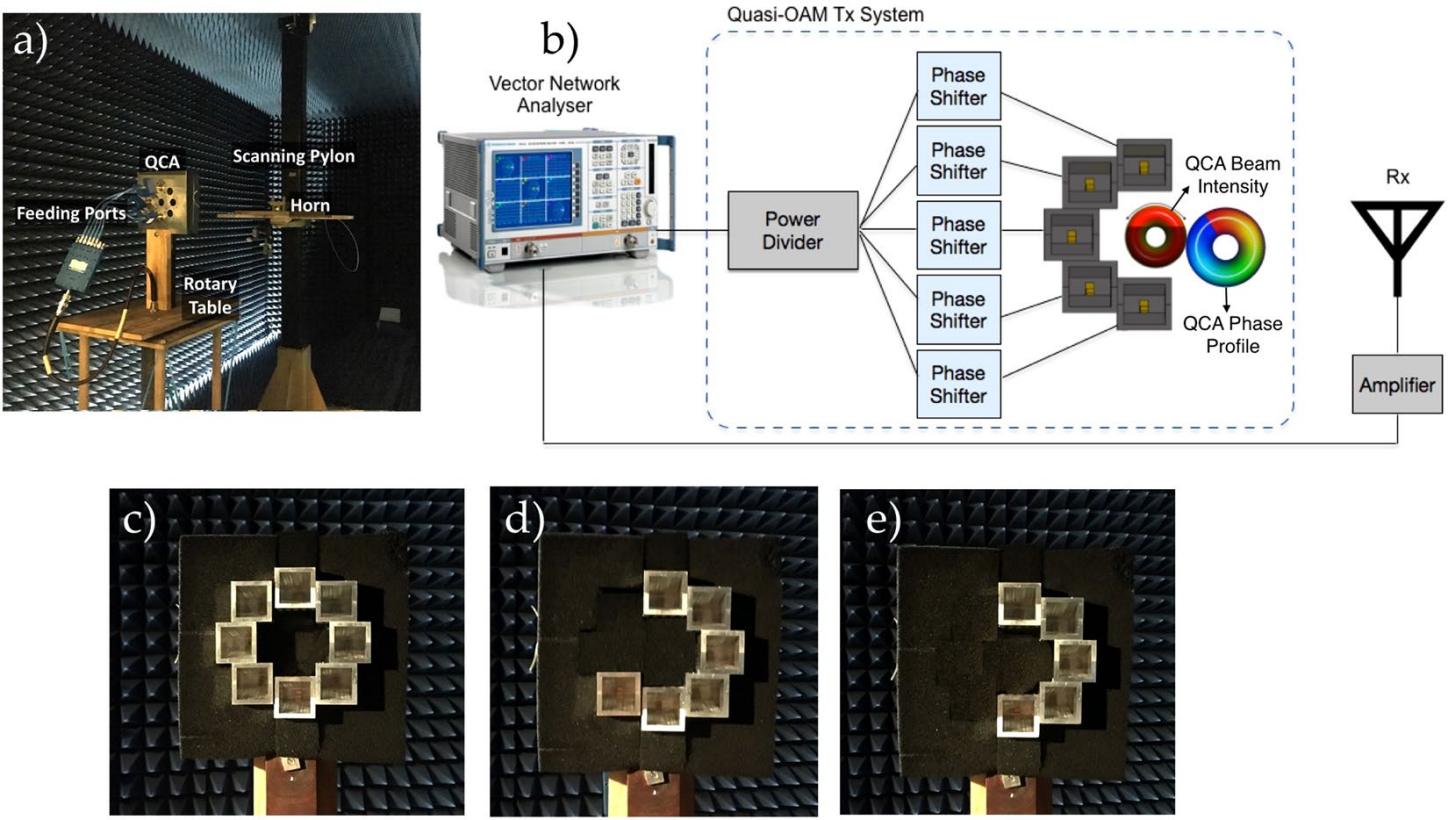
The measurement setup in the anechoic chamber is shown in (**a**), and the summary block diagram of the measurement configuration in (**b**). The Manufactured CA of *N* = 8 elements for whole OAM radiation pattern generation, and proposed QCA apertures of *N*_*Q*_ = 6 and *N*_*Q*_ = 5 for Quasi-OAM radiation pattern generation are shown in (**c**,**d**) and (**e**) respectively.

This section will outline the experimental setup and experimental results of our proposed Q-OAM generating system using the two proposed QCAs consisting of *N*_*Q*_ = 5, 6 elements, and a CA of *N* = 8 elements to generate whole OAM radiation patterns for comparison. The three arrays used in our experiments are shown in Fig. 7(c,d,e). Each array in Fig. 7(c,d,e) is mounted on a rotary table inside an anechoic chamber, rotating 360° for a cylindrical scan measurement. The QCA and CA arrays are used as the Q-OAM and whole OAM radiation pattern generators respectively.

#### Measured Radiation and Phase Patterns of proposed QCA and CA apertures

For comparison purposes, we will display the measured radiation pattern and phase pattern of a conventionally configured CA to produce whole OAM radiation patterns of the order, *l* = −1, +1 using the 8 element array CA shown in Fig. 7(c). Then the same will be displayed for QCA *N*_*Q*_ = 6 and QCA *N*_*Q*_ = 5 as shown in Fig. 7(d,e) respectively.

**Figure 8:**
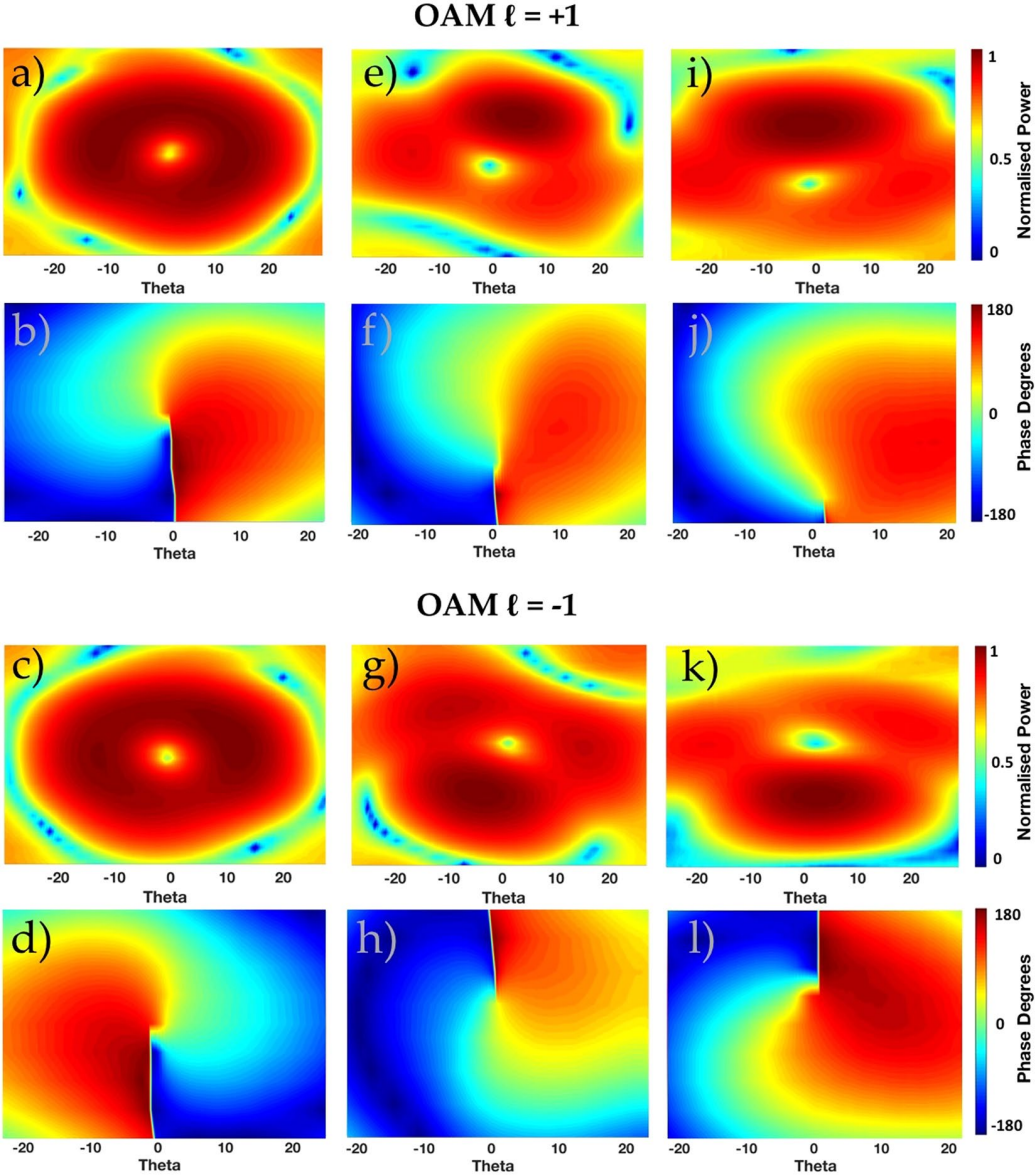
Measured OAM *l* = +1 radiation pattern and phase profile of CA *N* = 8 elements (**a**,**b**), QCA *N*_*Q*_ = 6 elements (**e**,**f**) and QCA *N*_*Q*_ = 5 elements (**i**,**j**) respectively. Measured OAM *l* = −1 radiation pattern and phase profile of CA *N* = 8 elements (**c**,**d**), QCA *N*_*Q*_ = 6 elements (**g**,**h**) and QCA *N*_*Q*_ = 5 elements (**k**,**l**) respectively.

As can be seen from Fig. 8(a,c), the radiation intensity pattern of a conventional CA of *N* = 8 elements exhibits a ‘donught’ shape radiation pattern with a null at boresight. This is a characteristic of all conventional OAM patterns, where the energy is distributed evenly around the azimuthal angle of the beam. Likewise, the phase pattern for OAM mode *l* = +1 shows a rotating phase front in the anticlockwise direction and clockwise for *l* = −1. For the radiation intensity patterns and phase profiles of our proposed QCA apertures to compare the generated OAM modes with the conventionally configured CA radiation patterns for OAM *l* = +1, −1, we display the radiation patterns for QCA of *N*_*Q*_ = 6 elements in Fig. 8(e–h) and QCA of *N*_*Q*_ = 5 elements in Fig. 8(i–l). It can be seen from the radiation patterns of the QCA with *N*_*Q*_ = 6 elements in Fig. 8(e,g) that the radiated beam follows the predicted beam direction from Eq. 11 and similar to that in our simulated results for both OAM modes *l* = −1, +1. Similar results are witnessed in the intensity pattern of the QCA of *N*_*Q*_ = 5 elements, where the measured radiation intensity is focused above and below the vortex region for OAM *l* = +1 and *l* = −1 respectively. The phase profiles of both QCA apertures in Fig. 8(f,h,j,l) both possess the characteristic of a rotating phase front associated with their respective OAM modes of *l* = +1 and *l* = −1. The area of highest intensity will therefore possess a Quasi-phase profile where the total change in phase in the main beam *δφ* = *β*, where *β* is the theoretical angle between two receiving probes for OAM orders *l* = +1, −1.

#### Measured Gain Increase of proposed QCA apertures vs. CA aperture

**Figure 9:**
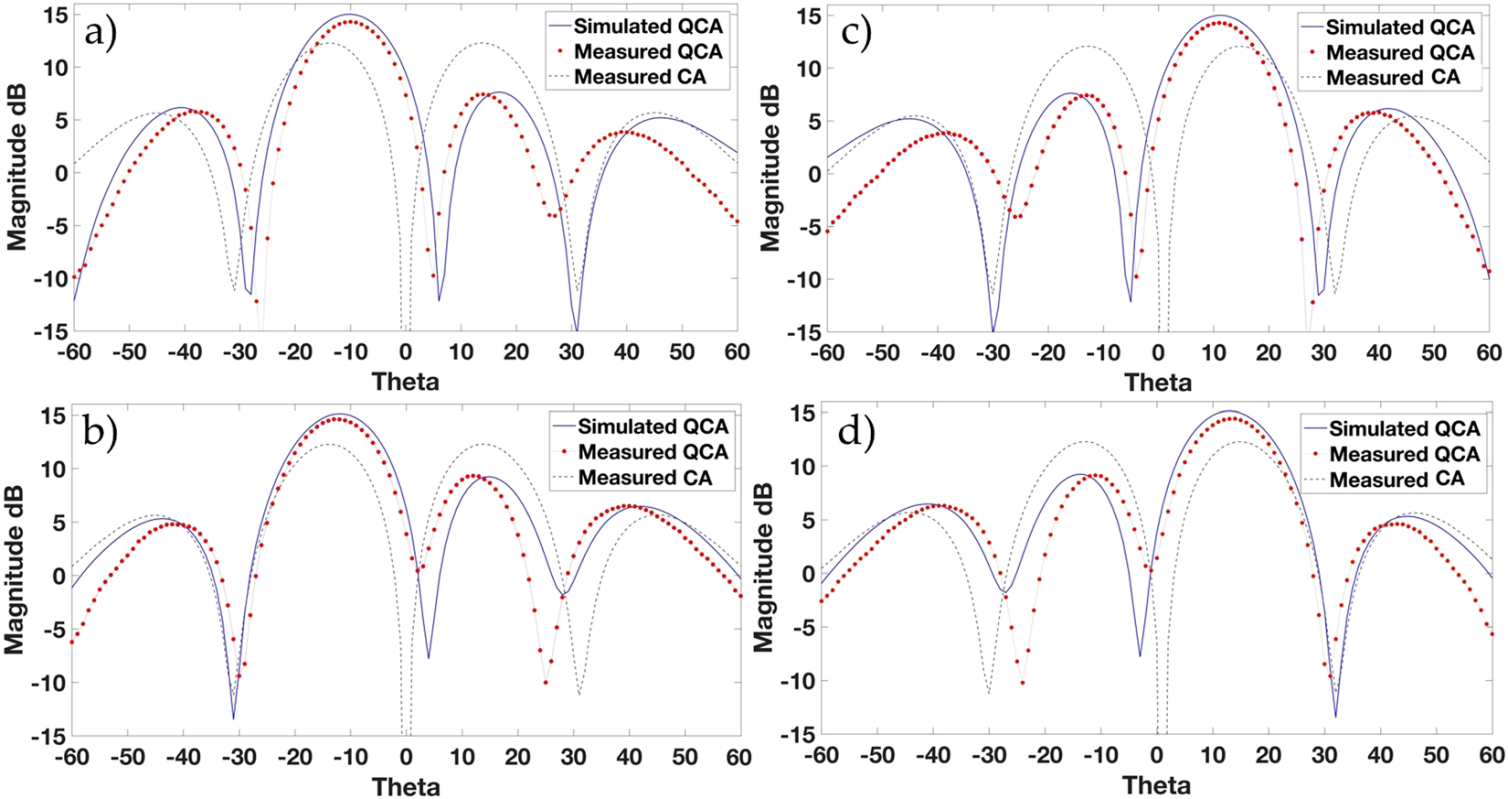
Measured OAM *l* = −1 and simulated radiation patterns for QCA of *N*_*Q*_ = 5 (**a**) and *N*_*Q*_ = 6 (**b**) elements vs. the measured conventionally configured CA of *N* = 8 elements. Measured OAM *l* = +1 and simulated radiation patterns for QCA of *N*_*Q*_ = 5 (**c**) and *N*_*Q*_ = 6 (**d**) elements vs. the measured conventionally configured CA of *N* = 8 elements.

The plots of the measured radiation patterns and gain of the proposed QCA apertures of *N*_*Q*_ = 5, 6 elements vs. the conventional CA of *N* = 8 elements are shown in Fig. 9. Each measured result is displayed with its simulated equivalent for the same OAM mode and array configuration, and measured against the measured gain of the conventionally configured CA to display the gain increase of each proposed QCA aperture and OAM mode. Small discrepancies between measured and simulated results are due to antenna and connector loss.

Measurement results in Fig. 9(a) show a 2 dB gain increase using QCA of *N*_*Q*_ = 5 elements and 2.2 dB using QCA of *N*_*Q*_ = 6 elements, as shown in Fig. 9(b) for mode order *l* = −1 as compared to CA of *N* = 8 elements. For mode order *l* = +1, Fig. 9(c,d) show an increase of 2.4 dB and 2.6 dB in gain for QCA of *N*_*Q*_ = 5 elements and QCA of *N*_*Q*_ = 6 elements respectively. It can also be noted that the divergence of the radiation patterns away from the propagation axis is less for QCA apertures as compared to CA conventional OAM radiation patterns. Although we have reduced the number of antennas typically used in an OAM generating circular array, we still witness an increase in gain as predicted by our theory and simulation results. This would deem the proposed Quasi-System of QCA apertures more suitable for longer range communications. However, where OAM multiplexing is used, the system may suffer from mode power decrease as discussed in the simulated gain results due to other modes being introduced with non-integer OAM modes. Further studies into the performance of the proposed Quasi-OAM generating apertures in a communication system with Bit-Error Rate (BER) measurements are envisioned to take place in future works for evaluation of the Quasi-OAM system’s robustness in comparison to conventional OAM communication systems.

## Conclusion

A proposed Quasi-OAM array is introduced in this paper to address some of the issues existing in conventional OAM circular arrays. These include pattern divergence, range and practicality for the physical antenna aperture size. By reducing the overall aperture size of circular arrays, while configuring the proposed QCA system in such a way to induce non-integer OAM carrying waves in the electromagnetic field, we demonstrated by simulations and experimental results that it is feasible to induce rotational linearly polarised OAM E-Fields similar to those induced by full circular arrays. This result illustrates that there is a means for reducing the need of a full circular array structure or having a full rotation of 360° in the feeding network to induce a full 360° rotation in the EM field associated with OAM modes *l* = +1 and *l* = −1. Using the theoretical rationale discussed in this paper for the reduction of destructive interference in an annular configuration, we have predicted and verified by simulation and experiments that we able to significantly reduce the aperture size and induce OAM E-field rotations associated with their dominant OAM mode *l* using the proposed Quasi-Circular Arrays. The predicted overall beam gain increase was also realised in simulation and experiment results, with an increase of the proposed QCA array’s gain by ~2.0 dB to 2.6 dB, as compared to a CA, while significantly reducing the aperture size and number of elements. The intended mode power generated however, is expected to decrease and pose as a challenge in OAM multiplexing systems, where the authors encourage further investigations into methods of OAM mode augmentation and cross-talk mitigation.

## Methods

Simulations of the proposed Q-OAM system were conducted using CST Microwave Studio for electromagnetic waves and antenna simulations. The simulation results were fixed at the operating frequency of 8.5 GHz, and the complete design of the radiating elements and proposed QCA arrays were rendered in the simulation environment before fabrication. For the Q-OAM experiments conducted and showcased in this paper, each array is connected to a power divider by equal length phase-matched cables and phase shifters connected to each element in the array to achieve the phase configurations discussed in this paper. The feed signal was produced by an Anritsu MS4644B Vector Network Analyser (VNA) which was divided into *N* parts, where *N*_*Q*_ = 5 for the QCA of 5 elements, *N*_*Q*_ = 6 for QCA of 6 elements and *N* = 8 for the conventional CA. The probing antenna used to measure the output of the three array apertures was a single back-launched horn antenna described in Fig. 3. This antenna was standardised against an ETS-Lindgren 3115 double ridged horn antenna. In the measurement setup, it was connected to an amplifier via the VNA. It was mounted on a scanning pylon and configured to cover 500 mm above boresight and 500 mm below in order to scan a 1 × 1 meter vertical plane. The measurements are taken in the far-field of the QCA and CA apertures based on the far-field criteria of *R*_*ff*_ = (2*D*^2^)/*λ*. The standardised antenna was positioned approximately 800 mm away from the arrays. Both the rotary table and scanning pylon were controlled by an external computer to complete a full cylindrical scan for each mounted array. A summary block diagram of the experimental setup is demonstrated in Fig. 7(b), and an image of the measurement setup in the anechoic chamber is captured in Fig. 7(a). The operating frequency for all measurements is kept the same as our simulation frequency, set to 8.5 GHz.

## Electronic supplementary material

Conventional CA of 8 Elements

Proposed QCA of 6 Elements

Proposed QCA of 5 Elements
