# Supplementary figures and images for: Quasi-Orbital Angular Momentum (Q-OAM) Generated by Quasi-Circular Array Antenna (QCA)
Source: Sci Rep. 2018 May 30;8:8363. doi: 10.1038/s41598-018-26733-6 (PMC5976661; doi:10.1038/s41598-018-26733-6)

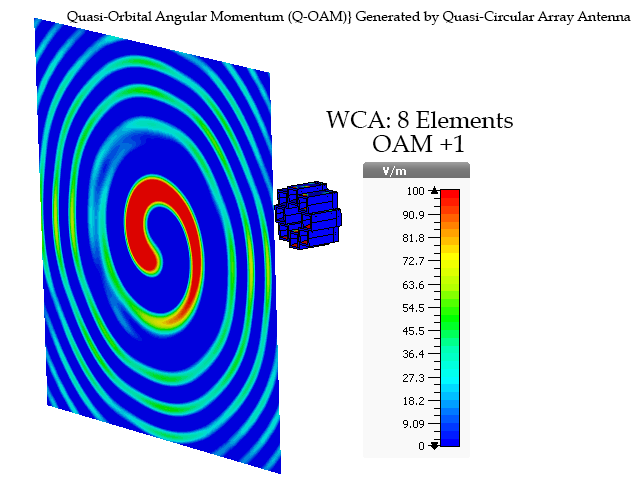

Supplement: Supplementary file 1 — Conventional CA of 8 Elements [file 41598_2018_26733_MOESM1_ESM.gif]

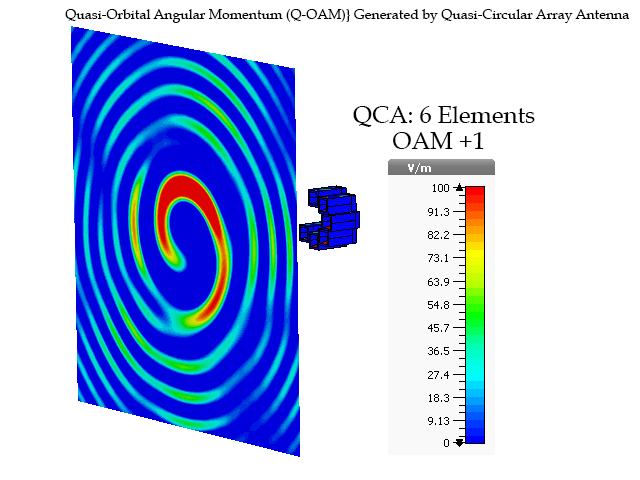

Supplement: Supplementary file 2 — Proposed QCA of 6 Elements [file 41598_2018_26733_MOESM2_ESM.gif]

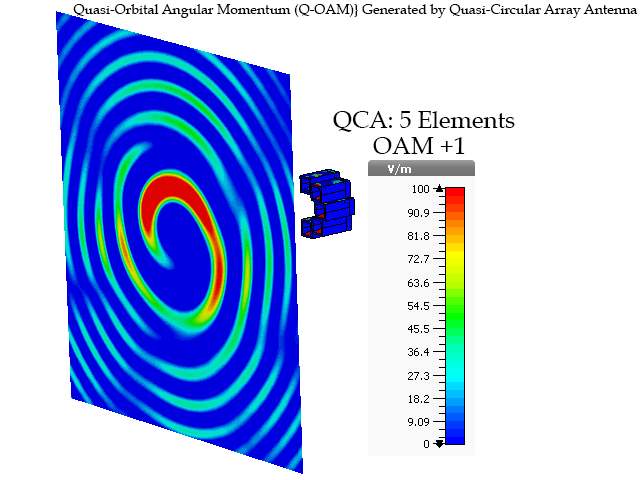

Supplement: Supplementary file 3 — Proposed QCA of 5 Elements [file 41598_2018_26733_MOESM3_ESM.gif]
